# Non-Coding Transcriptome Provides Novel Insights into the *Escherichia coli* F17 Susceptibility of Sheep Lamb

**DOI:** 10.3390/biology11030348

**Published:** 2022-02-22

**Authors:** Weihao Chen, Xiaoyang Lv, Weibo Zhang, Tingyan Hu, Xiukai Cao, Ziming Ren, Tesfaye Getachew, Joram M. Mwacharo, Aynalem Haile, Wei Sun

**Affiliations:** 1 College of Animal Science and Technology, Yangzhou University, Yangzhou 225009, China; 18552133709@163.com (W.C.); zhangwb7053@163.com (W.Z.); hty980105@163.com (T.H.); ziming970923@163.com (Z.R.); 2 Joint International Research Laboratory of Agriculture and Agri-Product Safety of Ministry of Education of China, Yangzhou University, Yangzhou 225009, China; dx120170085@yzu.edu.cn (X.L.); cxkai0909@163.com (X.C.); 3 International Centre for Agricultural Research in the Dry Areas, Addis Ababa 999047, Ethiopia; t.getachew@cgiar.org (T.G.); joram.mwacharo@sruc.ac.uk (J.M.M.); a.haile@cgiar.org (A.H.)

**Keywords:** *E. coli* F17, lamb, circRNA, miRNA, machine learning, ceRNA

## Abstract

**Simple Summary:**

Diarrhea and vomiting caused by *Escherichia coli* (*E. coli*) F17 are considered significant threats to animal farming. In the present study, RNA-Seq was performed to investigate the potential circRNA and miRNA biomarkers for *E. coli* F17-antagonism (AN) and -sensitive (SE) lambs. The results indicated that circRNA and miRNA expression is closely associated with the susceptibility of *E. coli* F17 in lambs. Numbers of circRNAs and miRNAs may serve as potential biomarkers for intestinal inflammatory response against *E. coli* F17 infection. Our study can provide a preliminary understanding of the underlying mechanisms of intestinal immunity.

**Abstract:**

It has long been recognized that enterotoxigenic *Escherichia coli* (ETEC) is the major pathogen responsible for vomiting and diarrhea. *E. coli* F17, a main subtype of ETEC, is characterized by high morbidity and mortality in young livestock. However, the transcriptomic basis underlying *E. coli* F17 infection has not been fully understood. In this study, RNA sequencing was performed to explore the expression profiles of circRNAs and miRNAs in the jejunum of *E. coli* F17-antagonism (AN) and -sensitive (SE) lambs. A total of 16,534 circRNAs and 271 miRNAs (125 novel miRNAs and 146 annotated miRNAs) were screened, and 214 differentially expressed (DE) circRNAs and 53 DE miRNAs were detected between the AN and SE lambs (i.e., novel_circ_0025840, novel_circ_0022779, novel_miR_107, miR-10b). Functional enrichment analyses showed that source genes of DE circRNAs were mainly involved in metabolic-related pathways, while target genes of DE miRNAs were mainly enriched in the immune response pathways. Then, a two-step machine learning approach combining Random Forest (RF) and XGBoost (candidates were first selected by RF and further assessed by XGBoost) was performed, which identified 44 circRNAs and 39 miRNAs as potential biomarkers (i.e., novel_circ_0000180, novel_circ_0000365, novel_miR_192, oar-miR-496-3p) for *E. coli* infection. Furthermore, circRNA-related and lncRNA-related ceRNA networks were constructed, containing 46 circRNA-miRNA-mRNA competing triplets and 630 lncRNA-miRNA-mRNA competing triplets, respectively. By conducting a serious of bioinformatic analyses, our results revealed important circRNAs and miRNAs that could be potentially developed as candidate biomarkers for intestinal inflammatory response against *E. coli* F17 infection; our study can provide novel insights into the underlying mechanisms of intestinal immunity.

## 1. Introduction

Diarrhea is the most commonly reported disease associated with infection by a complex mixture of bacteria in young animals. Among them, *Escherichia coli* (*E. coli*) is the major pathogenic bacterium responsible for diarrhea [1]. Pathogenic *E. coli* have been divided into five pathotypes based on the virulence properties and clinical signs of the host: enterotoxigenic *E. coli* (ETEC), enterohemorrhagic *E. coli* (EHEC), enteropathogenic *E. coli* (EPEC), enteroinvasive *E. coli* (EIEC), and diffusely enteroadherent *E. coli* (DAEC) [2].

Among these pathotypes, ETEC has been identified as the major agent of *E. coli*-related diarrhea [3,4,5,6]. ETEC adheres to intestinal epithelial cells (IECs), leading to the production and replication of enterotoxins [7]. Clinical reports revealed that ETEC infection exhibits enteropathogenicity, causing increased mortality and clinical signs such as severe vomiting and diarrhea [8]. The fimbrial adhesins, F5 [9], F17 [10], F18 [11], and F41 [12] are associated with ETEC mainly in young animals. *E. coli* F17, one of the main subtypes of ETEC, has been reported as the major pathogen associated with ETEC-related diarrhea worldwide, responsible for high morbidity and mortality [13,14,15]. The growing prevalence of *E. coli* F17 has renewed the sense of urgency for *E. coli* F17 research.

Following in the footsteps of high throughput sequencing technologies, myriad non-coding RNAs (ncRNA) were identified via RNA sequencing, such as long non-coding RNA (lncRNA), microRNA (miRNA) [16] and circular RNA (circRNA) [17]. Owing to their extensive participation in a variety of physiological and pathological processes, ncRNAs have received increasing attention in the past decade [18]. Emerging evidence has illustrated that circRNAs and miRNAs have regulatory roles in diverse farm animal diseases, particularly in mastitis [19,20], reproductive and respiratory syndrome [21,22], Marek’s disease [23,24], etc. In 2011, Salmena et al. [25] first proposed the “ceRNA hypothesis” as the letters of a new RNA language, describing the crosstalk within lncRNAs, circRNA, miRNAs, and mRNAs. To date, several lines of evidence have indicated that circRNAs and lncRNAs function as ceRNAs during *E. coli* infection. Yang et al. [26] reported that circ_2858 can increase *VEGFA* via sponging miR-93-5p during *E. coli* meningitis. In meningitic *E. coli*-caused blood-brain barrier disruption, LncRSPH9-4 modulates intercellular tight junctions via the miR-17-5p/*MMP3* axis [27]. In ETEC infection, several miRNAs have been confirmed to be a potential target for preventing pathogen infection; for example, miR-215 can regulate *E. coli* F18 resistance by targeting *EREG, NIPAL1*, and *PTPRU* [28]. In addition, miR-192 can reduce the adhesion ability of *E. coli* F18 and K88 in pig IECs via *DLG5* and *ALCAM* [29]. Nevertheless, the mechanisms of circRNAs and miRNAs in diarrhea caused by ETEC infection remain largely unknown, especially *E. coli* F17.

In the present research, RNA sequencing (RNA-seq) was performed to study the expression profiles of circRNAs and miRNAs in *E. coli* F17-antagonism and -sensitive lamb jejunum tissues. We undertook both bioinformatic and machine learning approaches to identify circRNA and miRNA biomarkers for *E. coli* F17 infection, and reveal the potential biological roles of them. Furthermore, we constructed ceRNA networks of circRNA-miRNA-mRNA and lncRNA-miRNA-mRNA. In summary, our results can provide a preliminary understanding of circRNAs and miRNAs in susceptibility of *E. coli* F17 in lambs, and promise to provide novel insight into intestinal immunity.

## 2. Material and Methods

### 2.1. Sample Collection

All experimental lambs were supplied by the Xilaiyuan Agriculture Co., Ltd. (Taizhou, China). *E. coli* F17-resistant and *E. coli* F17-sensitive lambs were detected from a challenge experiment of *E. coli* F17 (DN1401, fimbrial structural subunit: F17b, fimbrial adhesin subunit: Subfamily II adhesins, originally isolated from diarrheic calves) as described in our previous report [30]. 

Briefly, 50 healthy newborn lambs were randomly selected and reared on lamb milk replacer free of antimicrobial additives and free of probiotics from 1 day old to 3 days old. At 3 days after birth, lambs were divided into high-dose and low-dose challenge groups. Lambs in the high-dose and low-dose challenge groups were orally gavaged with 50.0 mL and 1.0 mL of actively growing culture of *E. coli* F17(1 × 10^9^ CFU/mL) for four days, respectively. Then, 10 healthy lambs in the high-dose challenge group and 10 lambs with severe diarrhea in the low-dose challenge group (evaluated via stool consistency scoring) were euthanized by administering pentobarbital overdose. Histopathological examination and bacteria plate counting of the intestinal contents were conducted to evaluate the severity of the diarrhea. Finally, six healthy lambs with mild intestinal pathology in the high-dose challenge group (antagonism group, AN) and six lambs with severe diarrhea in the low-dose challenge group (sensitive group, SE) with severe intestinal pathology were selected and proximal jejunum tissue was collected and snap-frozen in liquid nitrogen for RNA isolation. 

### 2.2. RNA Extraction and Sequencing

RNA was extracted from the jejunum tissue using TRIzol (Invitrogen, Carlsbad, CA, USA) per the manufacturer’s instructions. The quality of the extracted RNA was determined using an RNA Nano 6000 Assay Kit, and RNA integrity number (RIN) obtained using an Agilent 2100 Bioanalyzer with RIN ≥ 8.0 as the threshold.

The miRNA libraries were constructed using a NEBNext^®^ Multiplex Small RNA Library Prep Set for Illumina^®^ (NEB, Ipswich, MA, USA) per the manufacturer’s instructions. The miRNA libraries were sequenced on an Illumina HiSeq^TM^ 2500 platform with 50bp single-end reads strategy by Beijing Novogene Technology Co., Ltd. (Beijing, China).

The circRNA libraries were constructed using a NEBNext^®^ Ultra™ Directional RNA Library Prep Kit for Illumina^®^ (NEB, Ipswich, MA, USA) per the manufacturer’s instructions. The RNA libraries were sequenced on an Illumina HiSeq^TM^ 2500 platform with PE150 strategy (paired-end 150 bp) by Beijing Novogene Technology Co., Ltd.

Raw reads of FASTQ format were firstly obtained. Low-quality reads containing reads with adapters, reads with more than 10% N, and low-quality reads (quality scores <Q20; i.e., bases with sQ ≤ 5 more than 50% of all reads) were removed. Clean reads were generated and then mapped to the *Ovis aries* reference genome (*Oar_v4.0*) using Bowtie2 [31].

For known miRNA alignment, miRbase 20.0 was used as reference, and miRDeep22 [32] was used to assemble the miRNA transcripts. Then, srna-tools-cli was used to obtain the potential miRNA and draw the secondary structures; novel miRNA candidates from the transcripts were distinguished using miREvo [33] and miRDeep2 through exploring the secondary structure. The circRNA candidates from the transcripts were distinguished using find_circ [34] and CIRI2 [35].

### 2.3. Analysis of miRNA and circRNA Expression

The transcript per million (TPM) was used to estimate the expression levels of miRNA and circRNA candidates. Differentially expressed (DE) candidates were identified between AN and SE groups using DESeq R library (1.30.1) [36]. miRNAs and circRNAs were considered significantly DE as the threshold of corrected *p*-value (*p*-values adjusted by Benjamini and Hochberg’s approach) < 0.05.

### 2.4. Gene Ontology (GO) and Kyoto Encyclopedia of Genes and Genomes (KEGG) Functional Analyses

GO and KEGG enrichment were performed for the target genes of DE miRNAs (predicted using miRanda and RNAhybird) and source genes of DE circRNAs using GOseq R library (1.46.0) [37] and KOBAS (KO-Based Annotation System) programs [38], followed by a Fisher’s exact test with a false discovery rate (FDR) [39] multiple test correction to assess the statistical significance (*p* < 0.05).

### 2.5. Identification of Potential circRNA/miRNA Biomarkers for E. coli F17 Infection Using Machine Learning Methods

To identify potential lncRNA and mRNA biomarkers for *E. coli* F17 infection, a two-step machine learning approach (Random Forest-XGBoost, RX) combination Random Forest (RF) and Extreme Gradient Boosting (XGBoost) were performed. The randomForest R library (4.6.14) [40] and XGBoost R library (1.5.0.2) [41] were performed for the analyses. The detailed strategy for RX was described in our previous research [42].

Briefly, a range of parameters (Ntree and mtry for RF, colsample and eta for XGBoost) was systematically examined, and out-of-bag (OOB) error rate was calculated to determine the derive minimum hyperparameter values required for final analysis. For biomarkers identification, RF was firstly performed to select the subset of circRNAs and miRNAs with positive values of variable important measures (VIMs), then these selected circRNAs and miRNAs were further assessed by XGBoost. Similarly, XGBoost produces a VIM rank for the genes named “Gain”. In the current study, the VIM value of individual variable (circRNA or miRNA) denotes the relative contribution of the variable for each tree in the model; the higher the “Gain” value, the more important the variable is for generating a classification between lambs AN and SE lambs. Variables with a high “Gain” were, therefore, prioritized as potential circRNA/miRNA biomarkers for *E. coli* F17 infection.

### 2.6. Acquisition of lncRNA and mRNA Expression Dataset

The lncRNA and mRNA expression dataset used in this study was obtained from our previous research (unpublished data), which is available in the Sequence Read Archive (SRA) database under the study ID PRJNA759095. 

In brief, total RNA was extracted from jejunum tissue of six healthy lambs with mild intestinal pathology in the high-dose challenge group (antagonism group, AN) and six lambs with severe diarrhea in the low-dose challenge group (sensitive group, SE) with severe intestinal pathology as mentioned above. RNA libraries were sequenced using an Illumina HiSeq2500 equipment with the PE150 strategy. Reads were aligned to the *Ovis aries* reference genome (*Oar_v4.0*) using Hisat2 [43]. StringTie [44] was used to assemble the mRNA transcripts. Then, coding and non-coding RNA candidates from the transcripts were distinguished using Coding-Non-Coding-Index [45], Coded Potential Calculator-2 [46], and Pfam-scan [47]. Fragments Per Kilobase of transcript sequence per Million fragments sequenced (FPKM) was used to estimate the expression levels of candidate transcripts. DE lncRNAs and DE mRNAs were identified between AN and SE groups using edgeR R library (3.36.0, B LAB, Boston, MA, USA). lncRNAs and mRNAs were considered significantly DE as the threshold of corrected *p*-value (*p*-values adjusted by Benjamini and Hochberg’s approach) < 0.05. 

A total of 20,601 mRNAs and 12,426 lncRNAs were screened, within which 1465 DE mRNAs and 406 DE lncRNAs were identified between AN and SE lambs. Details can be found in Supplementary Table S1.

### 2.7. ceRNA Network Construction

The ceRNA networks was constructed on the basis of the co-expression association among mRNA, miRNA, circRNAs, and lncRNAs.

Based on all identified mRNAs, miRNAs, circRNAs, and lncRNAs, miRNA-related interaction pairs (miRNA-mRNA, miRNA-lncRNA, and miRNA-circRNA) were predicted using miRanda [48] and RNAhybrid [49]. Subsequently, interaction pairs sharing the same miRNAs were selected as candidate competing interactions for further analysis. Finally, Pearson correlation coefficient (PCC) and corrected *p*-value were calculated to estimate the co-expression relationship between circRNAs, lncRNAs, mRNAs, and miRNAs, and negatively miRNA-target pairs with PCC < −0.75 and corrected *p*-value < 0.05 (*p*-values adjusted by Benjamini and Hochberg’s approach) were selected to establish ceRNA networks of circRNA-miRNA-mRNA and lncRNA-miRNA-mRNA using cytoscape software (3.9.1) [50].

### 2.8. Validation of Sequencing Data

To validate the RNA-Seq data, 5 circRNAs and 5 miRNAs were randomly selected. The housekeeping genes *GAPDH* and U6 were selected as the reference genes. The sequences of the selected candidates and designed primers are shown in Supplementary Table S2.

Total RNA was extracted from the jejunum tissues from 12 lambs (six AN and six SE) and processed for sequencing using TRIzol (Invitrogen, Carlsbad, CA, USA) per the manufacturer’s instructions. The first strand of cDNA was prepared using FastKing gDNA Dispelling RT (Vazyme Biotech, Nanjing, Jiangsu, China) per the manufacturer’s instructions. The quality of the cDNA was evaluated by housekeeping gene amplification, and stored at −20 °C until use.

Real-time qPCR was performed in triplicate with cDNA to validate the reliability of RNA-Seq data. The 2^−ΔΔCt^ method [51] was used to calculate expression levels of selected circRNAs and miRNAs. The results were shown as relative expression level (log_2_FoldChange mean ± standard error) using GraphPad Prism 6 software.

## 3. Results

### 3.1. Overview of the Sequencing Data

Regarding the circRNA library, the average numbers of raw reads were 85,523,999 (AN) and 84,450,970 (SE); the average numbers of clean reads were 84,384,636 (AN) and 83,112,267 (SE); the average mapping rates for the AN and SE were 98.67% and 98.41%, respectively. Regarding miRNA library, the average numbers of raw reads were 13,862,992 (AN) and 13,415,602 (SE); the average numbers of clean reads were 13,490,636 (AN) and 13,091,889 (SE); the average mapping rates for the AN and SE were 97.19% and 97.49%, respectively. Detailed characteristics of the circRNA and miRNA libraries are shown in Table 1 and Table 2, respectively.

**Figure 1:**
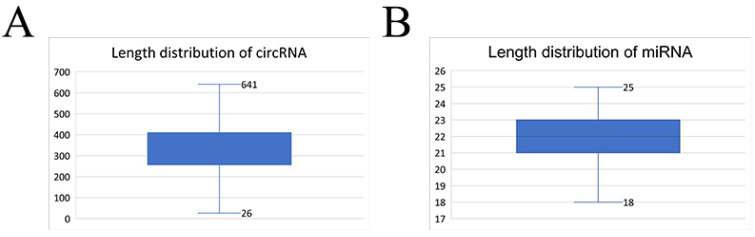
Length distribution of the identified circRNAs (**A**) and miRNAs (**B**).

Based on the results of CIRI2, miREvo, and miRDeep22, we identified a total of 16,534 circRNAs and 271 miRNAs; 125 of the miRNAs were novel and 146 were annotated miRNAs. Most of the circRNAs were 200–400 nt long, with an average length of 334.28 nt (Figure 1A), whereas most of the miRNAs were 200–400 nt long, with an average length of 21.74 nt (Figure 1B).

### 3.2. Differentially Expressed circRNAs and miRNAs

TPM was performed to estimate the expression levels of circRNAs and miRNAs; miRNAs had a relatively higher expression than that of circRNAs, and the expression of circRNAs and miRNAs were similar between AN lambs and SE lambs (Supplementary Figure S1).

**Figure 2:**
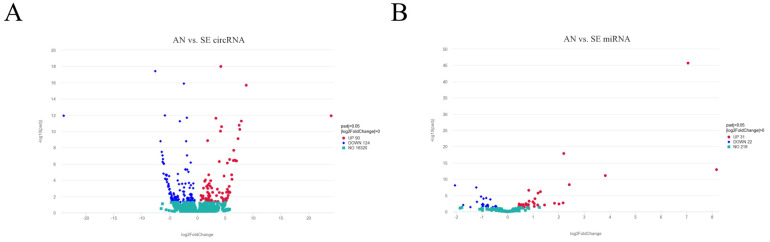
Volcano plot of differentially expressed (DE) circRNAs (**A**) and DE miRNAs (**B**).

We identified 214 DE circRNAs between the AN and SE libraries, within which 90 were upregulated and 124 downregulated (Figure 2A). We also identified 53 DE miRNAs between the AN and SE libraries, within which 31 were upregulated and 22 downregulated (Figure 2B). Detailed results are provided in Supplementary Table S3.

### 3.3. Functional Analysis

**Figure 3:**
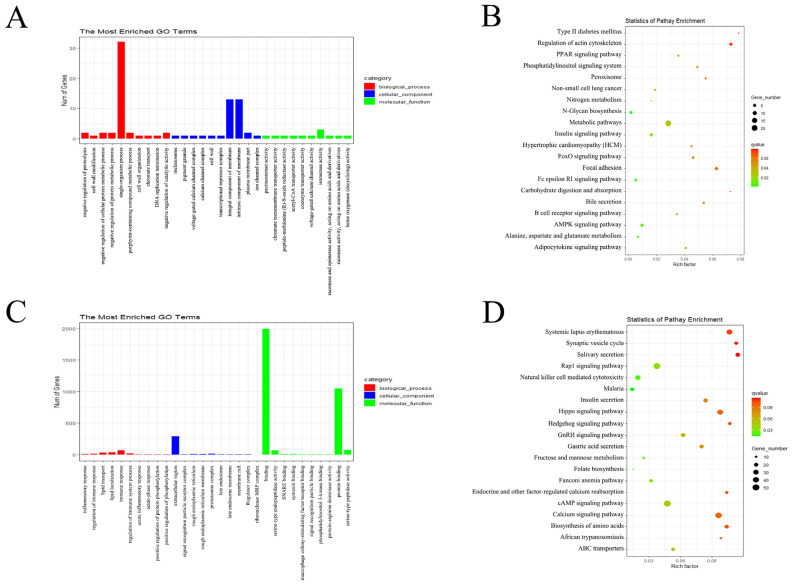
Top annotated GO terms (**A**) and top enriched KEGG pathways (**B**) of source genes of DE circRNAs. Top annotated GO terms (**C**) and top enriched KEGG pathways (**D**) of target genes of DE miRNAs.

GO and KEGG enrichment analyses were conducted using source genes of DE circRNAs and target genes of DE miRNAs. Figure 3 shows some of the top enriched GO terms and KEGG pathways; detailed enrichment analyses results can be seen in Supplementary Table S4.

The source genes of DE circRNAs were significantly enriched in 61 GO terms. The top enriched GO terms were single-organism process (GO:0044699), isomerase activity (GO:0016853), and membrane (GO:0016020) in biological process (BP), molecular function (MF), and cellular component (CC), respectively. The source genes of DE circRNAs were significantly enriched in 14 KEGG pathways, within which pathways related to intestinal inflammation were enriched, such as PPAR signaling pathway (oas03320) and N-Glycan biosynthesis (oas00510).

The target genes of DE miRNAs were significantly enriched in 132 GO terms. The top enriched GO terms were phosphorylation (GO:0016310), binding (GO:0005488), and extracellular region (GO:0005576) in biological process (BP), molecular function (MF), and cellular component (CC), respectively. The target genes of DE miRNAs were significantly enriched in 7 KEGG pathways, within which pathways related to intestinal inflammation were enriched, such as natural killer cell mediated cytotoxicity (oas04650) and Rap1 signaling pathway (oas04015).

### 3.4. Potential circRNA/miRNA Biomarkers for E. coli F17 Infection

The final parameters used for RF and XGBoost analyses of miRNA and circRNA expression datasets were chosen based on a systematic evaluation of a range of values, details of which can be seen in Supplementary Table S5.

**Figure 4:**
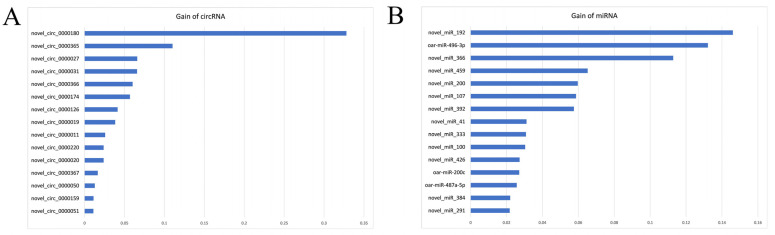
Gain value of top circRNAs (**A**) and miRNAs (**B**) selected by Random Forest-XGBoost.

For circRNA biomarker identification, 2437 circRNAs with positive VIM values were identified by RF, then 44 circRNAs were further selected by XGBoost (Figure 4A). The top three circRNAs with highest Gain values were novel_circ_0000180 (0.33), novel_circ_0000365 (0.11), and novel_circ_0000027 (0.07).

For miRNA biomarker identification, 68 miRNAs with positive VIM values were identified by RF, then 39 miRNAs were further selected by XGBoost (Figure 4B). The top three miRNAs with highest Gain values were novel_miR_192 (0.15), oar-miR-496-3p (0.13), and novel_miR_366 (0.11).

### 3.5. ceRNA Network

**Figure 5:**
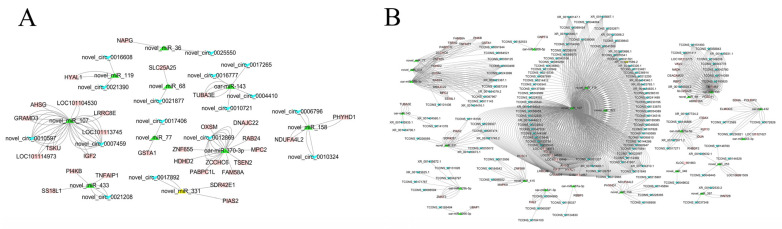
ceRNA networks of circRNA-miRNA-mRNA (**A**) and lncRNA-miRNA-mRNA (**B**), where the “V” shape (blue), triangle (blue), and rectangle (red) represent circRNAs (lncRNAs), miRNAs, and mRNAs, respectively.

From the results of miRanda and RNAhybrid, combined with calculated PCC and corrected *p*-value, we identified 79 miRNA-mRNA pairs, 47 miRNA-circRNA pairs, and 347 miRNA-lncRNA pairs. Then, ceRNA networks were constructed based on the interaction pairs with shared miRNAs. We finally obtained 46 circRNA-miRNA-mRNA competing triplets among 30 mRNAs, 10 miRNAs, and 16 circRNAs (Figure 5A); and 630 lncRNA-miRNA-mRNA (Figure 5B) competing triplets among 44 mRNAs, 23 miRNAs, 137 lncRNAs, details of which can be seen in Supplementary Table S6.

For a better understanding of the huge and complicated ceRNA networks, we calculated the connections of each node in the network. Notably, the same topmost connected regulator was identified in the two ceRNA networks: a novel miRNA named novel_miR_107, which was found to participate in 18 circRNA-miRNA-mRNA competing triplets and 386 lncRNA-miRNA-mRNA competing triplets. Our results suggested that novel_miR_107 may serve as a star competing endogenous biomarker for *E. coli* F17 infection. The Hi-res ceRNA networks can be seen in Supplementary Figures S1 and S2.

### 3.6. Validation of Sequencing Data

**Figure 6:**
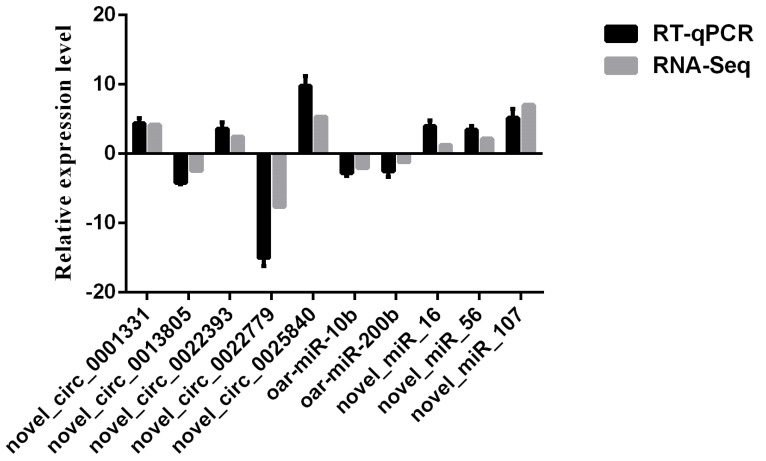
Comparisons of the results of the RNA–seq and RT–qPCR analyses of selected circRNAs and miRNAs.

The comparison of the expression level of circRNAs and miRNAs selected for verification of the accuracy of sequencing between RNA-Seq and RT-qPCR are shown in Figure 6. The results indicated that selected circRNAs and miRNAs showed similar expression patterns between RNA-Seq and RT–qPCR, suggesting the reliability of our sequencing data.

## 4. Discussion

In our previous research, we studied the transcriptomic characteristics of lamb spleen in response to *E. coli* F17 infection and revealed numbers of DE mRNAs, circRNAs, and lncRNAs [30,52]. However, as the first barrier against *E. coli* F17, the transcriptomic roles of the intestine in the process of *E. coli* F17 infection have not been well documented. In the present study, by integrating transcriptomic and multiple bioinformatic approaches, we provide a preliminary understanding of the transcriptomic profiles of circRNAs and miRNAs in *E. coli* F17-resistant (AN) and *E. coli* F17-sensitive (SE) lamb jejunum. 

In the present study, we identified a total of 16,534 novel circRNAs, 125 novel miRNAs, and 146 annotated miRNAs. The number of identified circRNAs was remarkably higher than previously identified in the spleen [52]; similar results were also obtained in the circRNA-seq study in clave jejunum [53] and porcine intestinal epithelial cells [54]. Over the past decade, studies of circRNAs were mainly focused on brain tissue [55]; our results suggest that circRNAs are also highly enriched in the intestine, which suggests that the intestine could be an important tissue to investigate. 

By applying DEseq, we detected 214 DE circRNAs and 53 DE miRNAs, indicating clearly different expression profiles of circRNAs and miRNAs between the AN and SE lambs. The most upregulated DE circRNAs (ranked by fold change and padj) was novel_circ_0025840, whose source gene is transmembrane protein 27 (*TMEM27*), a crucial regulator produced in beta cells and linked to beta cell proliferation [56]. The most downregulated DE circRNAs was novel_circ_0022779. Interestingly, the source gene of novel_circ_0022779 is transmembrane protein 16E (*TMEM16E*, also known as Anoctamin 5, *ANO5)*, which is also a member of the transmembrane protein family and plays a role in regenerative muscle repair [57]. Although the functions of these circRNAs are largely unclear, our results suggest that they may serve as the principal regulators during *E. coli* F17 infection, and might function together with transmembrane protein family members. The most upregulated DE miRNAs was a novel miRNA, namely, novel_miR_107; not much is known about the novel miRNA, but the high expression of novel_miR_107 in AN lambs suggest that novel_miR_107 would make a prime candidate for future research. The most downregulated DE miRNAs was miR-10b, one of the most upregulated miRNAs in human cancers and strongly expressed in highly metastatic cancer cells [58,59]. In the present study, miR-10b was highly expressed in SE lambs. Considering the role of miR-10b in the cancer cell cycle, migration, and invasion [60,61], miR-10b may play an important role in intestinal immunity by regulating the cell progress of *E. coli* F17 infected-IECs. Of course, in-depth work is needed to confirm this possibility.

To further understand the function of the DE circRNAs and miRNAs, we performed GO and KEGG enrichment analyses using source genes of DE circRNAs and target genes of DE miRNAs. GO enrichment analysis showed target genes of DE miRNAs were mainly involved in the immune response, including inflammatory response, regulation of immune response, and regulation of immune system process. Source genes of DE circRNAs were primarily involved in diverse cellular processes such as cell wall modification, negative regulation of cellular protein metabolic process, and cell wall organization. Similar results were also obtained in the KEGG pathway enrichment analysis: target genes of DE miRNAs were mainly involved in the immune-response-related pathways, such as natural killer cell mediated cytotoxicity (early defenses against cells undergoing various forms of stress such as infection with bacteria and viruses [62,63]) and ABC transporter (primarily import systems of *E. coli*, [64,65]). Source genes of DE circRNAs were mainly involved in metabolic-related pathways, such as N-Glycan biosynthesis, Alanine, aspartate and glutamate metabolism, and nitrogen metabolism. Taken together, our results suggest that DE miRNAs may be the principal regulators of intestinal inflammatory response, and DE circRNAs may function against *E. coli* F17 infection through cellular metabolic pathways. It is worth noting that several well-studied *E. coli* infection-related pathways, such as TLR and NF-kappaB pathways, were not enriched in our study; one potential explanation for these inconsistencies is that all experimental lambs were challenged with *E. coli* F17 in our study, while these pathways were initially revealed between challenged and unchallenged individuals.

Machine learning (ML) methods have shown promising results in identifying biologically important genes when applied to transcriptomic datasets [66,67,68,69]. In our previous research, a comparison of the classification accuracy of decision-tree-based ML methods (Random Forest, XGBoost) and DE analysis methods (edgeR, t-test) was conducted, and we found that a combination method of Random Forest and XGBoost (RX) outperformed the other four methods (Random Forest, XGBoost, t-test, and edgeR) with the highest classification accuracy [42]. Hence, RX was performed in the present study to identify potential circRNA/miRNA biomarkers for *E. coli* F17 infection. Forty-four circRNAs and 39 miRNAs were finally selected by RX, within which the circRNA and miRNA with the highest Gain values were novel_circ_0000180 and novel_miR_192; the specific roles of these novel candidates in *E. coli* infection have not yet been revealed. The high Gain values demonstrated that they achieved a good performance in distinguishing AN and SE lambs in our transcriptomic datasets; in addition, the decision-tree-based strategy underlying RX [70] also indicated that certain interactivity exists between them and other important biomarkers picked by RX. There is a high probability that these circRNAs and miRNAs can act as key regulators in *E. coli* F17 infection, and thus assist in discovering novel regulatory mechanisms associated with intestinal immunity.

To uncover the ceRNA crosstalk underlying intestinal inflammatory response against *E. coli* F17 infection, we constructed ceRNA networks of circRNA-miRNA-mRNA and lncRNA-miRNA-mRNA. A total of 46 circRNA-miRNA-mRNA competing triplets and 630 lncRNA-miRNA-mRNA competing triplets were identified. Within these, several regulators have been demonstrated to be involved in various disease processes, such as miR-370-3p (cholangiocarcinoma, [71], acute myeloid leukemia [72], oral squamous carcinoma [73], hepatocellular carcinoma [74], ovarian cancer [74]), miR-143 (DE between the duodenum of *E. coli* F18 -sensitive and resistant weaned piglets [75]), miR-133 (acute myocardial infarction [76], breast cancer [77]), and *MAPK9* (production of inflammation mediator [78]). In addition, several novel regulators were also found to participate in many ceRNA competing triplets, such as novel_miR_107, novel_miR_119, novel_miR_433, and TCONS_00072826. The most connected regulator was a novel miRNA, namely, novel_miR_107; novel_miR_107 participated in 18 circRNA-miRNA-mRNA competing triplets and 386 lncRNA-miRNA-mRNA competing triplets. Of note, novel_miR_107 was also a potential miRNA biomarker with high Gain value selected by RX; these results can further demonstrate the biological value of RX in RNA-seq analysis.

## 5. Conclusions

In summary, our study presented expression profiles of circRNAs and miRNAs in *E. coli* F17-antagonism and -sensitive lamb jejunum tissues. A total of 214 DE circRNAs and 53 DE miRNAs were identified between the AN and SE lambs, and a series of integrated bioinformatic analyses revealed several potential important circRNAs (i.e., novel_circ_0000180, novel_circ_0022779, and novel_circ_0025840) and miRNAs (i.e., novel_miR_107, miR-10b, and novel_miR_192). Moreover, we constructed circRNA-related and lncRNA-related ceRNA networks involved in intestinal inflammatory response against *E. coli* F17 infection. The findings from this study can help elucidate the molecular mechanisms underlying intestinal immunity.

## Figures and Tables

**Table 1:** Summary of the circRNA library.

Sample	Raw Reads	Clean Reads	Mapping Rate (%)	Error Rate (%)	Q20 (%)	Q30 (%)	GC Content (%)
AN1	86,448,964	85,310,470	98.68	0.03	97.55	93.27	51.32
AN2	82,985,976	82,314,372	99.19	0.03	97.00	91.76	46.32
AN3	81,095,934	79,701,960	98.28	0.03	97.49	93.16	51.61
AN4	94,502,330	93,722,960	99.18	0.03	97.25	92.49	48.07
AN5	84,496,940	83,246,004	98.52	0.03	97.45	93.06	50.25
AN6	83,613,850	82,012,052	98.08	0.03	97.49	93.19	54.43
SE1	82,325,980	81,420,394	98.90	0.03	97.31	92.67	52.18
SE2	83,101,628	81,439,640	98.00	0.03	97.39	93.00	48.07
SE3	83,731,304	82,241,834	98.22	0.03	97.45	93.09	49.79
SE4	80,794,124	79,478,658	98.37	0.03	96.90	91.99	56.07
SE5	92,174,900	90,902,860	98.62	0.03	97.35	92.99	49.55
SE6	84,577,884	83,190,218	98.36	0.03	96.54	91.14	49.89

Note: AN and SE represent antagonism group and sensitive group, respectively. Error rate% represents overall sequencing error rate. Quality score (Q) represent the probability of incorrect based call.

**Table 2:** Summary of the miRNA library.

Sample Name	Raw Reads	Clean Reads	Clean Bases	Error Rate (%)	Q20 (%)	Q30 (%)	GC Content (%)
AN1	16,273,383	16,059,313	98.68	0.01	99.49	98.16	49.67
AN2	13,434,545	13,274,363	98.81	0.01	99.50	98.35	48.56
AN3	14,558,297	14,136,725	97.10	0.01	99.06	96.65	48.87
AN4	11,883,680	11,545,885	97.16	0.01	99.10	96.96	49.54
AN5	15,402,425	15,008,710	97.44	0.01	99.04	96.79	49.09
AN6	11,625,621	10,918,820	93.92	0.01	99.30	97.26	50.01
SE1	18,148,953	17,949,815	98.90	0.01	99.49	98.30	48.85
SE2	13,392,060	13,198,054	98.55	0.01	99.32	97.92	49.46
SE3	10,839,760	10,594,527	97.74	0.01	99.34	97.74	49.80
SE4	13,718,249	13,297,138	96.93	0.01	99.02	97.04	49.09
SE5	12,498,474	11,906,416	95.26	0.01	98.97	96.90	50.58
SE6	11,896,114	11,605,384	97.56	0.01	99.33	97.73	48.81

Note: AN and SE represent antagonism group and sensitive group, respectively. Error rate% represents overall sequencing error rate. Quality score (Q) represents the probability of incorrect based call.

## Data Availability

The circRNAs and miRNAs datasets presented in this study can be found in online repositories. The names of the repository/repositories and accession number(s) can be found below: https://www.ncbi.nlm.nih.gov/ (accessed on 8 January 2022), PRJNA786953.

## Supplementary Materials

The following supporting information can be downloaded at: https://www.mdpi.com/article/10.3390/biology11030348/s1, Figure S1: TPM distribution of the identified circRNAs (A) and miRNAs (B). TPM distribution of the identified circR-NAs (C) and miRNAs (D) between AN lambs and SE lambs; Figure S2: Hi-res ceRNA networks of circRNA-miRNA-mRNA; Figure S3: Hi-res ceRNA networks of lncRNA-miRNA-mRNA; Table S1: Differentially expressed analysis results of mRNAs and lncRNAs; Table S2: Sequence and designed primers of selected circRNAs and miRNAs; Table S3: Differentially expressed analysis results of circRNAs and miRNAs; Table S4: GO and KEGG enrichment results of source genes of DE circRNAs and target genes of DE miRNAs; Table S5: Parameter training results and detailed results of RF and RX, Table S6: Competing triplets of circRNA-miRNA-mRNA and lncRNA-miRNA-mRNA.
