# Supplementary material for: Non-Coding Transcriptome Provides Novel Insights into the Escherichia coli F17 Susceptibility of Sheep Lamb
Source: Biology (Basel). 2022 Feb 22;11(3):348. doi: 10.3390/biology11030348 (PMC8945857; doi:10.3390/biology11030348)
Supplement: Supplementary file 1 [file biology-11-00348-s001.zip › Supplementary Figure S1.pdf]

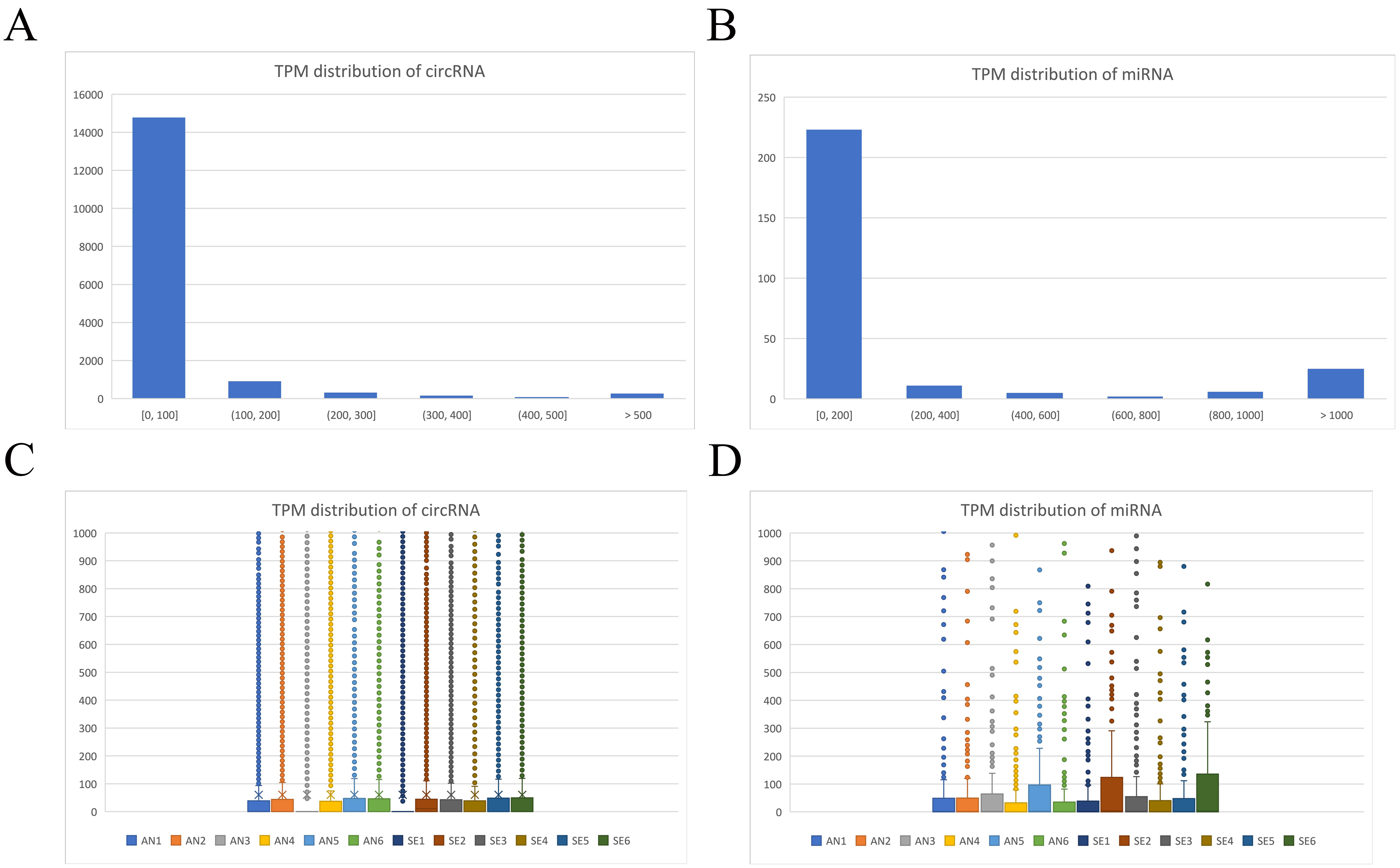

Figure S1. TPM distribution of the identified circRNAs (A) and miRNAs (B). TPM distribution of the identified circR-NAs (C) and miRNAs (D) between AN lambs and SE lambs.
