# Supplementary material for: Non-Coding Transcriptome Provides Novel Insights into the Escherichia coli F17 Susceptibility of Sheep Lamb
Source: Biology (Basel). 2022 Feb 22;11(3):348. doi: 10.3390/biology11030348 (PMC8945857; doi:10.3390/biology11030348)
Supplement: Supplementary file 1 [file Supplementary Figure S3.pdf › Supplementary Figure S3.pdf]

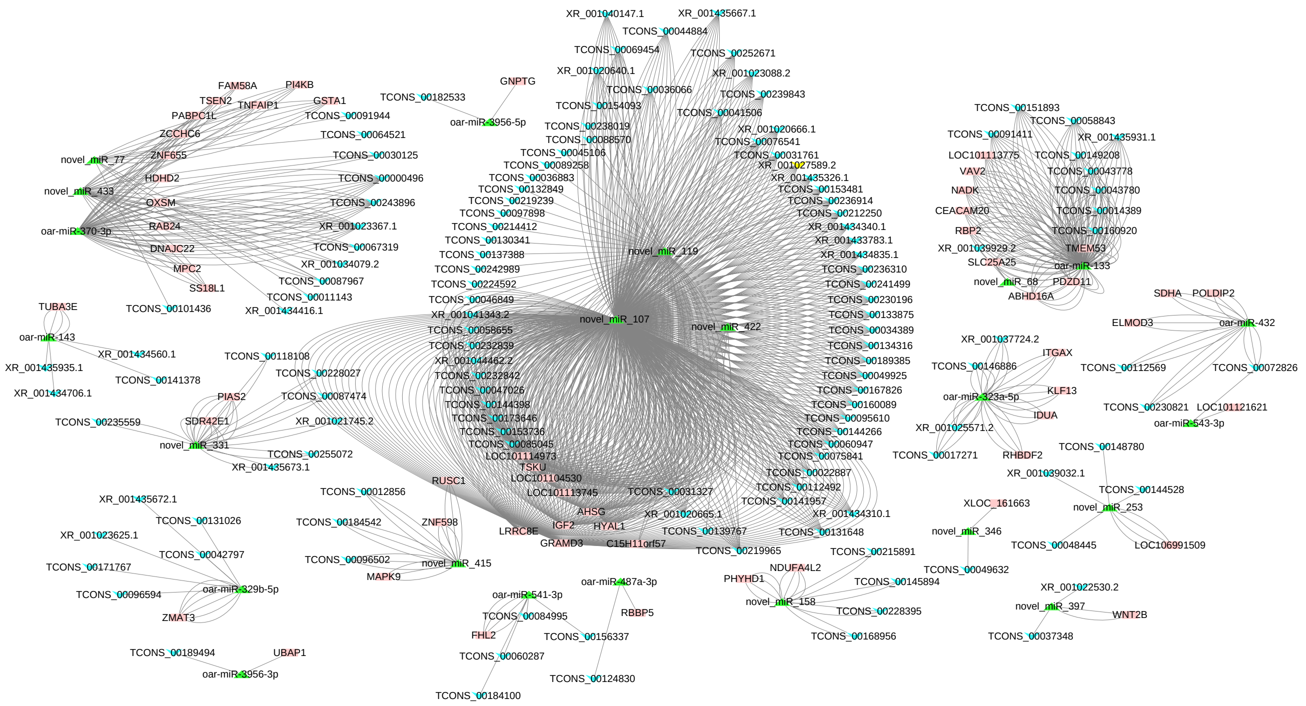

Figure S3. Hi-res ceRNA networks of lncRNA-miRNA-mRNA

Note: the “V” shape(blue), triangle (blue), and rectangle (red) represent lncRNAs, miRNAs, and mRNAs, respectively.
